# Supplementary material for: IL-33 drives group 2 innate lymphoid cell-mediated protection during Clostridium difficile infection
Source: Nat Commun. 2019 Jun 20;10:2712. doi: 10.1038/s41467-019-10733-9 (PMC6586630; doi:10.1038/s41467-019-10733-9)
Supplement: Supplementary file 1 — Supplementary Information [file 41467_2019_10733_MOESM1_ESM.pdf]

Supplementary Material

IL-33 drives group 2 innate lymphoid cell-mediated protection during *Clostridium difficile* infection.

Frisbee *et al.*

Supplementary Figure 1

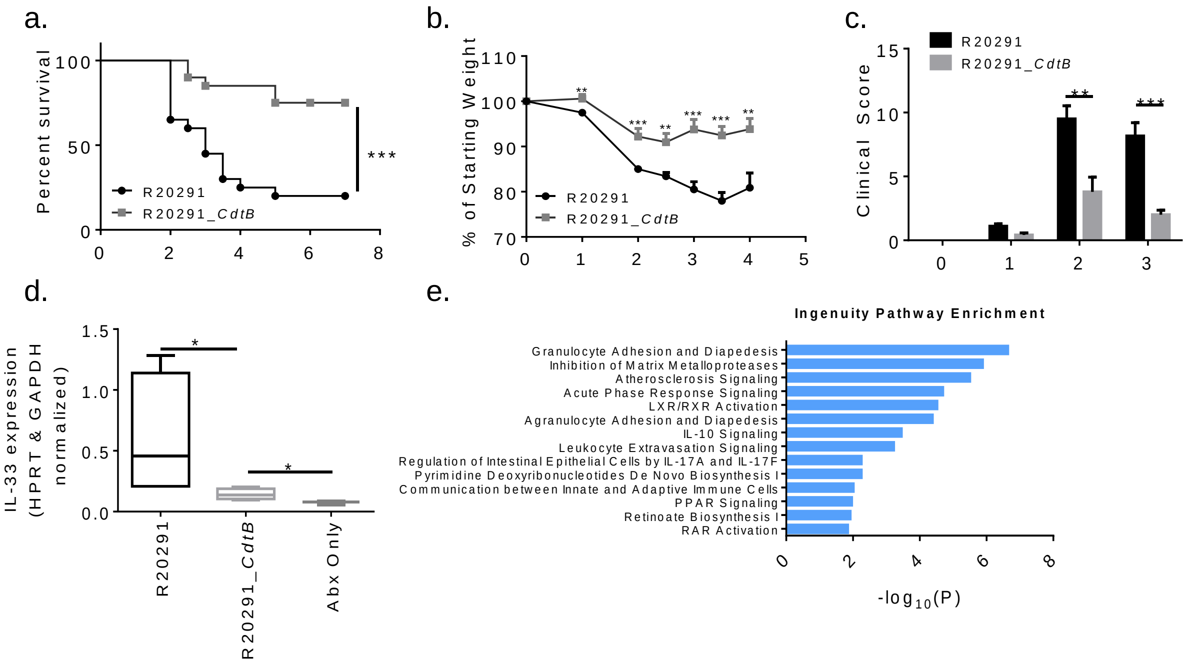

Supplementary Figure 1. IL-33 transcripts, are upregulated in response to severe CDT(+) and attenuated CDT(-) *C. difficile* infection.

(***a-c***) Mice were infected with R20291 (CDT+) or R20291_*cdtb (CDT-)* and (*a*) survival, (***b***) body-weight and (***c***) clinical scores were assessed on each day post infection. (***d***) IL-33 transcript abundance in infected caecal tissue was measured (Day 3) by Taqman qPCR normalized to housekeeping genes. Abx= antibiotic treated. (***e***) Enriched pathways of the top upregulated transcripts (log FC > 0.5) listed by significance (-log10(p)) and created using the Ingenuity Database. Data are presented as means ± SEM (***a–c***) *n* = 10 per experiment and combined from 2 independent infections and (***d***) *n* = 4,4,3 animals per group representative of 3 independent experiments. (***a***) Comparison made by log rank test. (***b-c)*** Comparison made by student *t* test. Statistical significance is demarked as *P < 0.05, **P < 0.01, and ***P < 0.001. Error bar indicates SEM.

Supplementary Figure 2

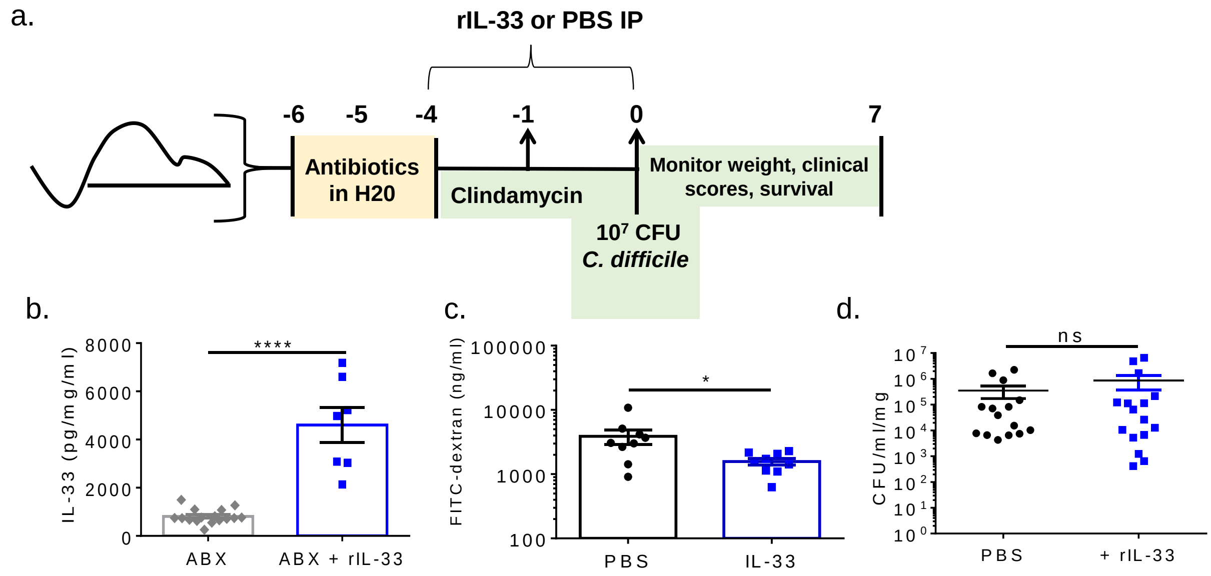

Supplementary Figure 2. IL-33 treatment protects the gut epithelial barrier during infection but does not decrease translocating commensals into the liver.

(***a***) IL-33 treatment experimental design. (***b***) Total IL-33 protein present in caecal tissue with and without IL-33 treatment measured by ELISA. Abx=antibiotic treated. (***c***) FITC-dextran concentration present in serum after gut permeability assay during infection (Day 2). (***d***) IL-33 and PBS treated mice were infected with R20291 *C. difficile* liver tissue was harvested and colony forming units were counted on BHI agar plates (Day 2). (***b,c***) Comparison made by student t test (***b***, *n* =16,7; ***c***, *n* = 9 and combined from 2 independent experiments). (***d***) Comparisons for commensal burden were made by Mann–Whitney U tests (*n*=15,16 combined from 2 independent experiments). Statistical significance is demarked as *P < 0.05, **P < 0.01, and ***P < 0.001. Error bar indicates SEM.

Supplementary Figure 3

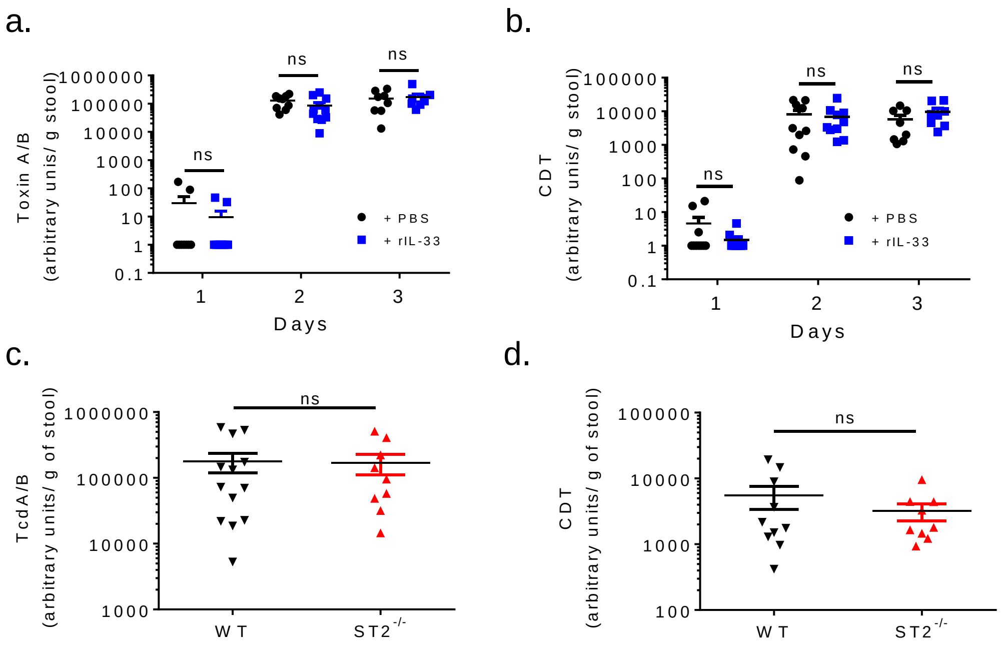

Supplementary Figure 3. Exogenous and endogenous IL-33 do not alter C. difficile toxin levels during infection.

(***a,b***) IL-33 treated (blue square) vs vehicle treated (black circles) mice were sacrificed on day 1, 2 and 3 post infection and (***a***) Toxins A/B in the caecal contents were assessed by ELISA and (***b***) CDT in the caecal contents was assessed by ELISA. (***c,d***) ST2$^{-/-}$ vs. WT mice were sacrificed on day 2 post infection and (***c***) Toxins A and B in the caecal contents were assessed by ELISA and (***d***) CDT in the caecal contents was assessed by ELISA. Comparisons for toxins were made by student t test and Mann–Whitney U tests. (***a***, *n*= 9,11; ***a***, *n*= 10, ***c***&***d***, *n*=13,9 independent animals per group). Data representative of 2 independent experiments. Statistical significance is demarked as *P < 0.05, **P < 0.01, and ***P < 0.001. Error bar indicates SEM.

Supplementary Figure 4

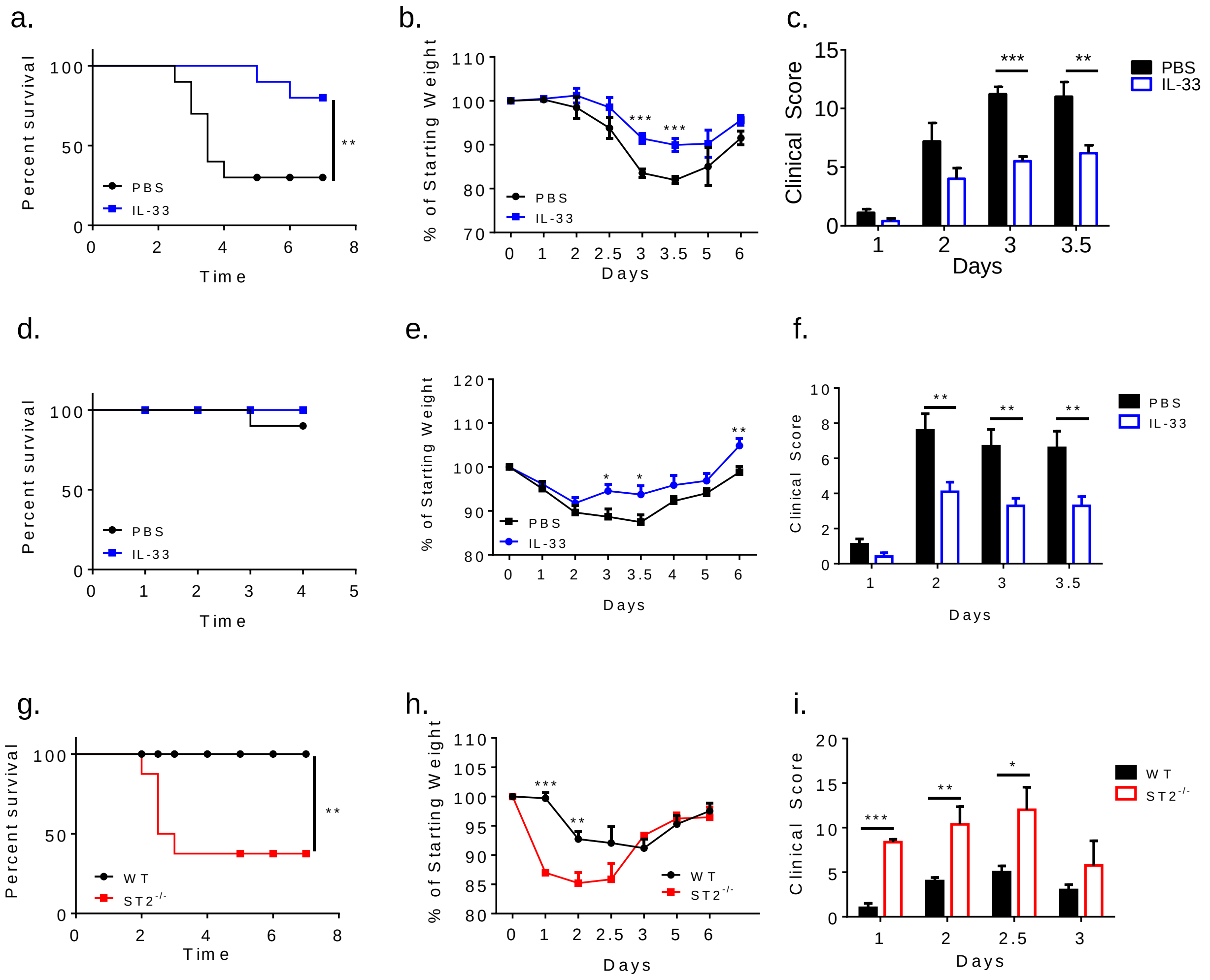

Supplementary Figure 4. IL-33 protects from mortality, weight-loss and clinical scores in non-027 (VPI 10643) and non-CDT, R20291_cdtb infection.

(***a-f***) Wildtype mice treated with IL-33 or with vehicle control were infected with (***a-c***) VPI 10643, or (***d-f***) attenuated mutant, R20291_cdtb (CDT-) and (***a,d***) survival, (***b,e***) body-weight and (***c,f***) clinical scores were assessed on each day post infection. (***g-i***) ST2$^{-/-}$ mice and WT controls were infected with the attenuated mutant, R20291_cdtb and (***g***) survival, (***h***) body-weight and (***i***) clinical scores were assessed on each day post infection. (***a–i***) combined *n* = 10,12 independent animals per group and (***a,d,g***) comparison made by log rank test. (***b,c,e,f,h,i***) Comparison made by student t test. Statistical significance is demarked as *P < 0.05, **P < 0.01, and ***P < 0.001. Error bar indicates SEM.

Supplementary Figure 5

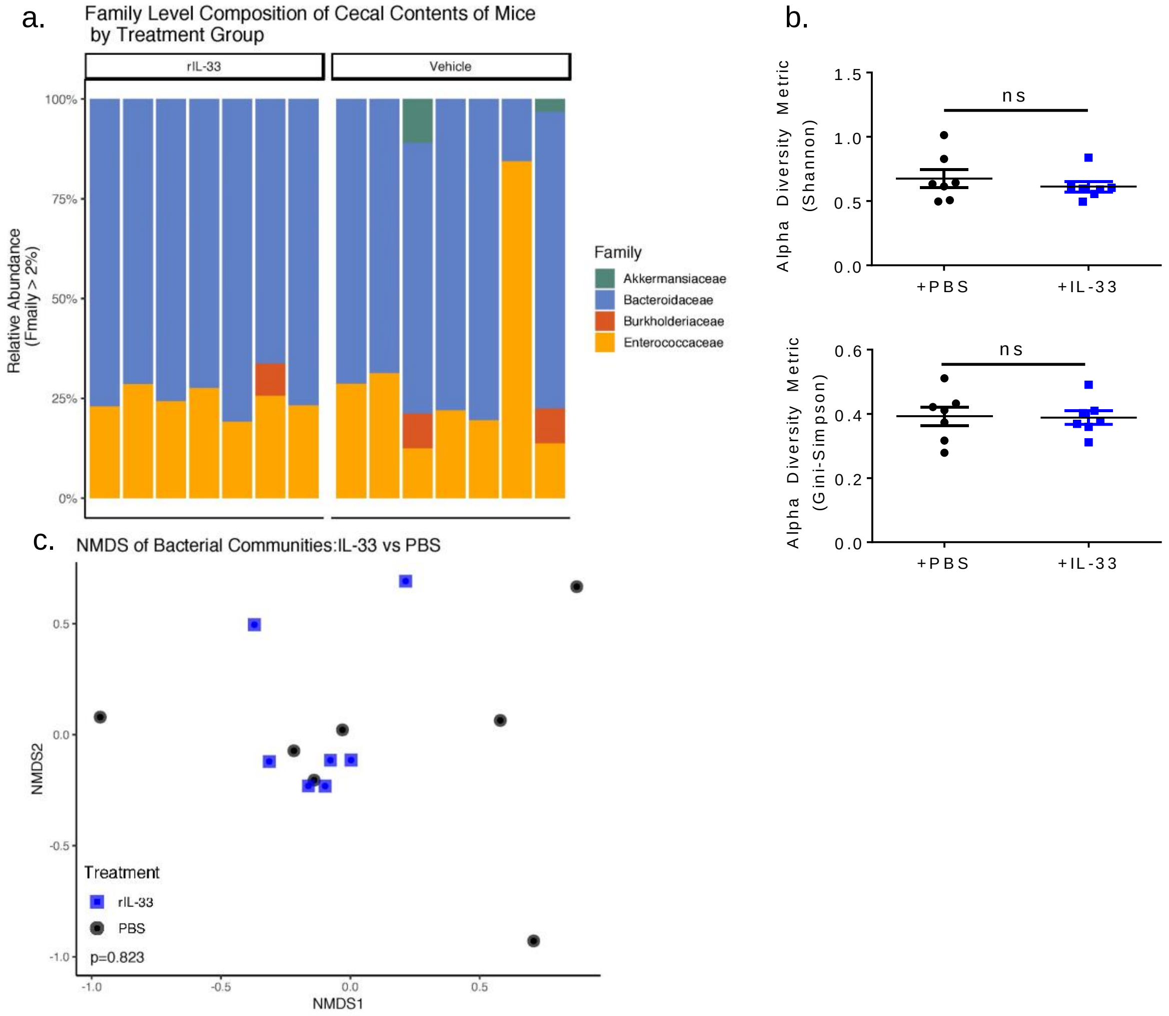

Supplementary Figure 5. IL-33 treatment does not significantly alter the microbiota community prior to infection.

Mice were treated with IL-33 or vehicle control, in addition to broad-spectrum antibiotic cocktail used in infection model. (***a***) Bar plot of relative phylum abundance of IL-33 vs. control groups prior to infection with *C. difficile*. (***b***) Microbiota diversity in caecal contents of untreated donors and antibiotic treated FMT recipients using Shannon (upper) and Simpson index (lower). (***c***) NMDS plot of Bray-Curtis dissimilarity index from caecal samples of IL-33 and vehicle mice post antibiotic treatment and before C. difficile infection. (***b***) Comparison made by ANOVA test (n =7 independent mice per group). (***c***) Comparisons made using permutational multivariate analysis of variance (PERMANOVA). Statistical significance is demarked as *$P < 0.05$, **$P < 0.01$, and ***$P < 0.001$. Error bar indicates SEM.

Supplementary Figure 6

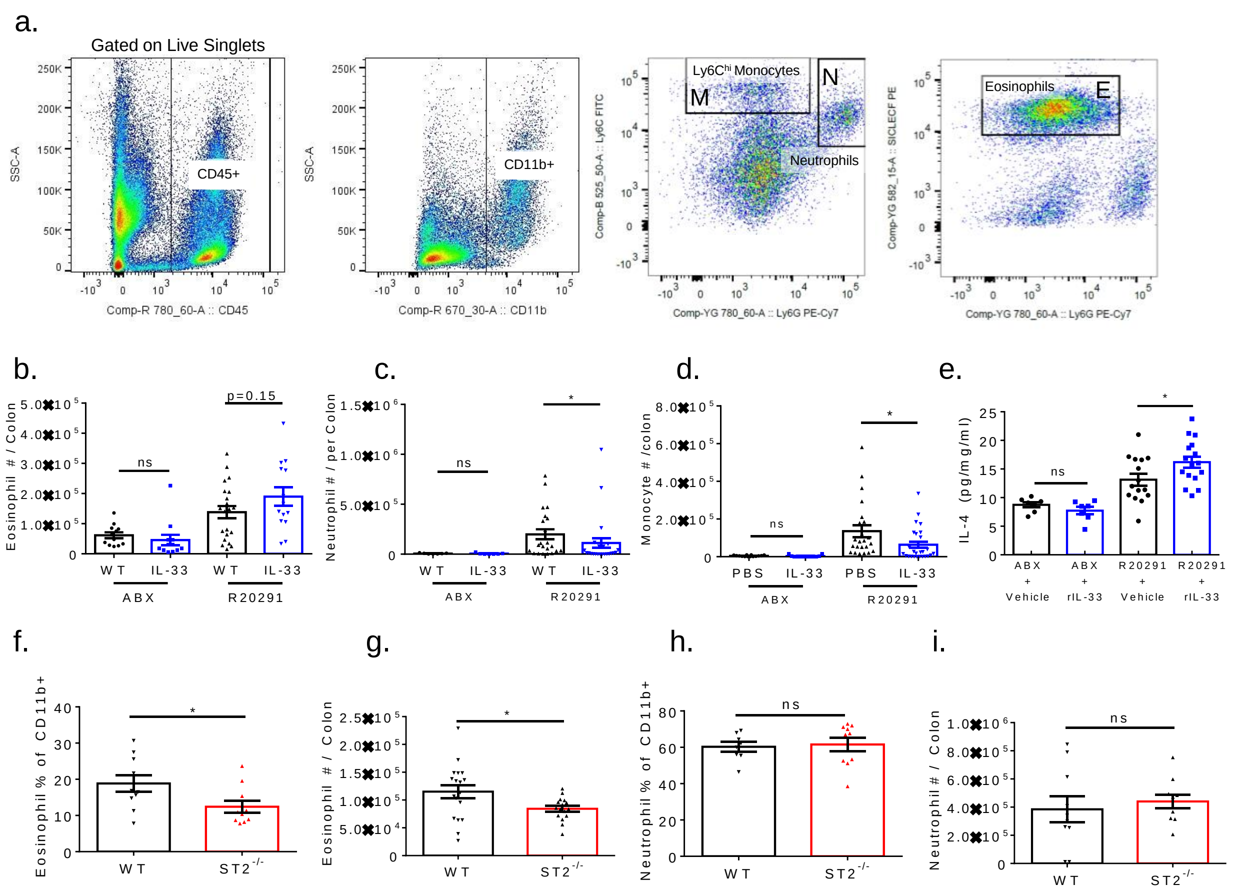

Supplementary Figure 6. IL-33 regulates type-17 vs. type-2 immunity during CDI.

(***a***) Gating strategy for flow cytometry characterization of E= eosinophils ($CD45^+$ $CD11b^+$ $SiglecF^+$ $Ly6g^-$), N= neutrophils ($CD45^+$ $CD11b^+$ $Ly6g^+$ $Ly6c^+$), and M= $Ly6C^{hi}$ monocytes ($CD45^+$ $CD11b^+$ $Ly6g^-$ $Ly6c^+$) in the colon. Representative dot plot from an IL-33 treated mouse during R20291 infection (Day 2). (***b-d***) Total number of (***b***) Eosinophils, (***c***) Neutrophils (***d***) $Ly6C^{hi}$ Monocytes in IL-33 vs. vehicle treated mice during infection or in uninfected mice, antibiotic (ABX) treated mice. (***e***) IL-4 expression in gut caecal tissue with and without IL-33 treatment in infected and uninfected mice. (***f-i***) $ST2^{-/-}$ mice and wildtype mice were infected with R20291 and (***f***) eosinophil frequency (% of CD11b+), (***g***) eosinophil number, (***h***) neutrophil frequency (% of CD11b+) and (***i***) neutrophil number were assessed by flow cytometry (Day 2). Comparisons between groups made by two-tailed student *t* test or Mann-Whitney test as described in methods (***b***, *n*= 12,12,21,14; ***c***, *n*= 7,7,22,24; ***d***, *n*= 14,13,8,8; ***e***, *n*= 7,7,15,16; ***f-i***, *n*=10 mice per group and combined from 2 independent experiments). Data combined from 2 independent experiments. Statistical significance is demarked as $^*P < 0.05$, $^{**}P < 0.01$, and $^{***}P < 0.001$. Error bar indicates SEM.

Supplementary Figure 7

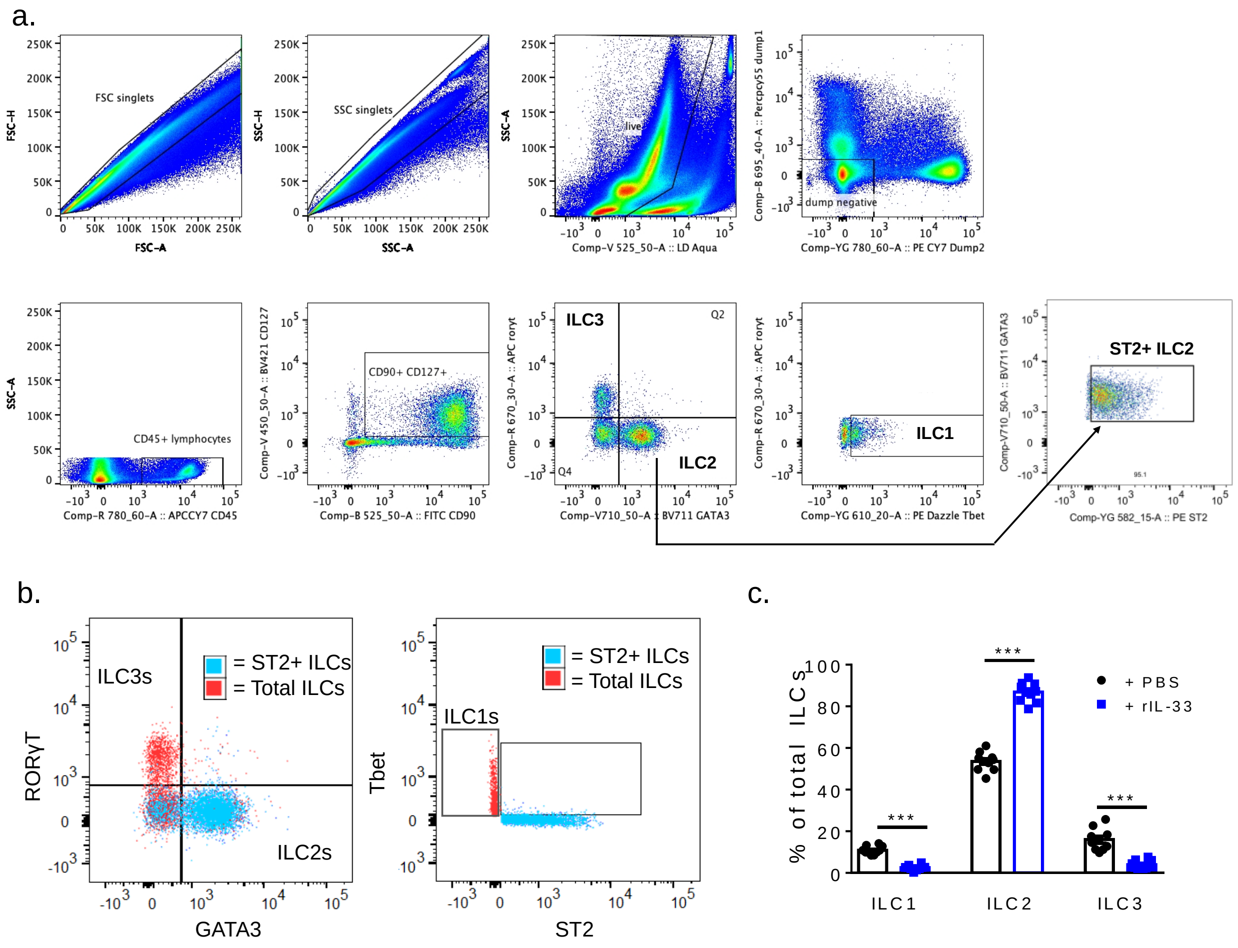

Supplementary Figure 7. IL-33 treatment expands ILC2s that co-express the IL-33 receptor (ST2) during *C. difficile* infection.

(***a-c***) Immune profiling of ILCs within the colon of IL-33 treated or untreated mice during R20291 infection by flow cytometry. (***a***) Flow cytometry gating strategy of ILCs within the colon. Lineage cells (CD19+, CD3+, CD5+, CD11c+, CD11b+, FcεR+) were gated out, then ILC subsets were further identified by the following strategy: ILC1 (Lin^- $CD45^+$ $CD90^+$ $CD127^+$ $Tbet^+$); ILC2 (Lin^- $CD45^+$ $CD90^+$ $CD127^+$ $GATA3^+$ $ST2^+$); ILC3 (Lin^- $CD45^+$ $CD90^+$ $CD127^+$ $RoryT^+$. (***b***) ILC2s (Lin- $CD45^+$ $CD90^+$ $CD127^+$ $GATA^+$) co-express the ST2 receptor (blue). (***c***) Frequency of ILC1s, ILC2s, and ILC3s within the colon of IL-33 treated or untreated mice infected with R20291 (Day 2). ***(c)*** Groups compared with student-t tailed t test (*n*=10 representative of 3 independent experiments). Statistical significance is demarked as *P < 0.05, **P < 0.01, and ***P < 0.001. Error bar indicates SEM.

Supplementary Figure 8

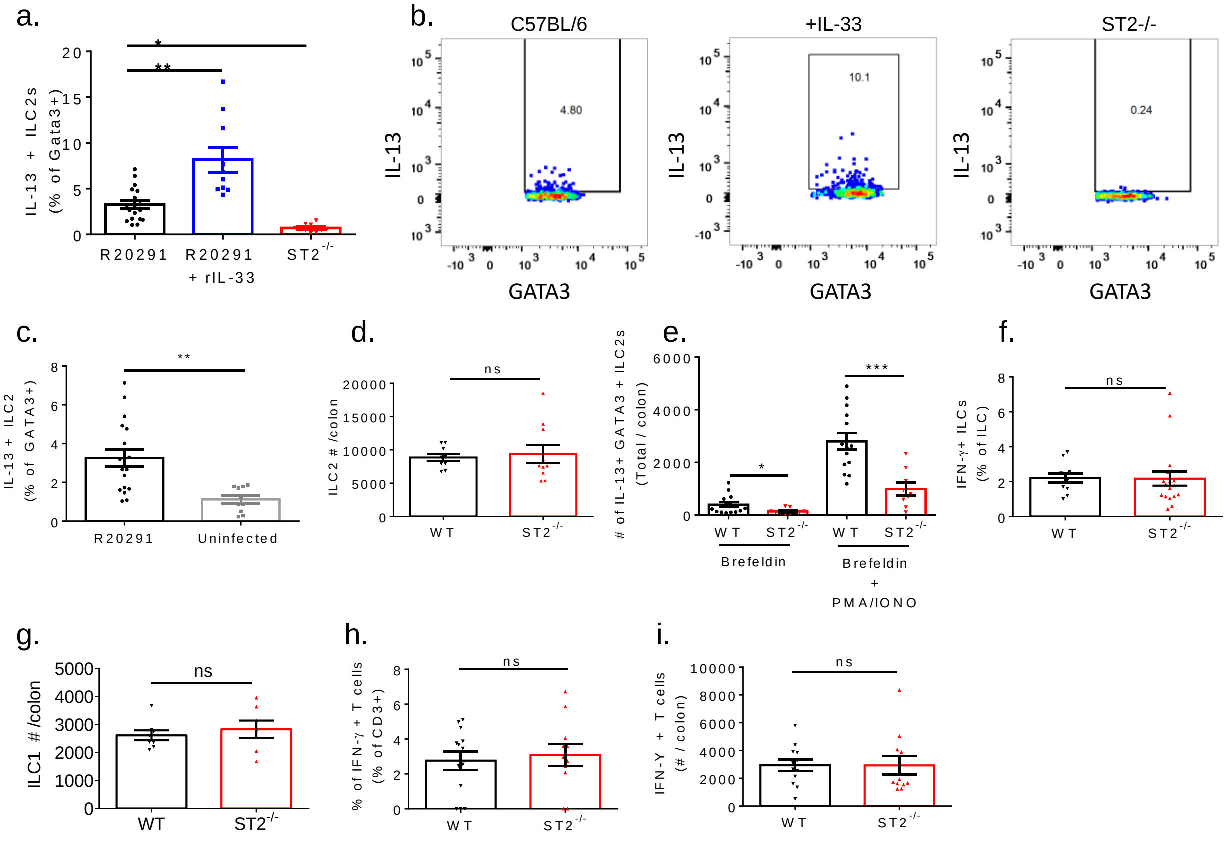

Supplementary Figure 8. IL-33 activates IL-13+ ILC2s during *C. difficile* infection.

(***a-e***) Immune profiling of IL-13+ GATA3+ ILC2s within the colon of infected IL-33 treated, ST2$^{-/-}$, WT, or uninfected mice by flow cytometry. (***a***) % of IL-13 + ILC2s in *C. difficile* infected WT, IL-33 treated, or ST2$^{-/-}$ mice (Day 2). (***b***) Representative flow plots of IL-13 expression during R20291 infection. (***c***) % of ILC2s in R20291 infected vs. uninfected mice. (***d***) Total number of ILC2s and (***e***) IL-13+ ILC2s in the colon during infection. ***(f-i***) Immune profiling type-1 immunity during infection. ***(f)*** Frequency of IFN-γ+ ILC1s and (***g***) Number of T-bet+ ILC1s in the colon during ST2$^{-/-}$ vs. WT infection. (***h***) Frequency of IFN-γ+ CD3+ T cells and (***i***) Number of IFN-γ+ T cells in the colon during infection. (***a***) Groups compared with ANOVA (n=18,10,10 animals from two combined independent experiments). (***c-i***) Groups compared by student-t tailed t test and Mann Whitney test (***c***, *n*= 18, 10; ***e***, *n*=14,10,14,10; ***g***, *n*=7; ***c,e,g,h***, *n*= 10,11) Combined from 2 independent experiments. Statistical significance is demarked as *P < 0.05, **P < 0.01, and ***P < 0.001. Error bar indicates SEM.

Supplementary Figure 9

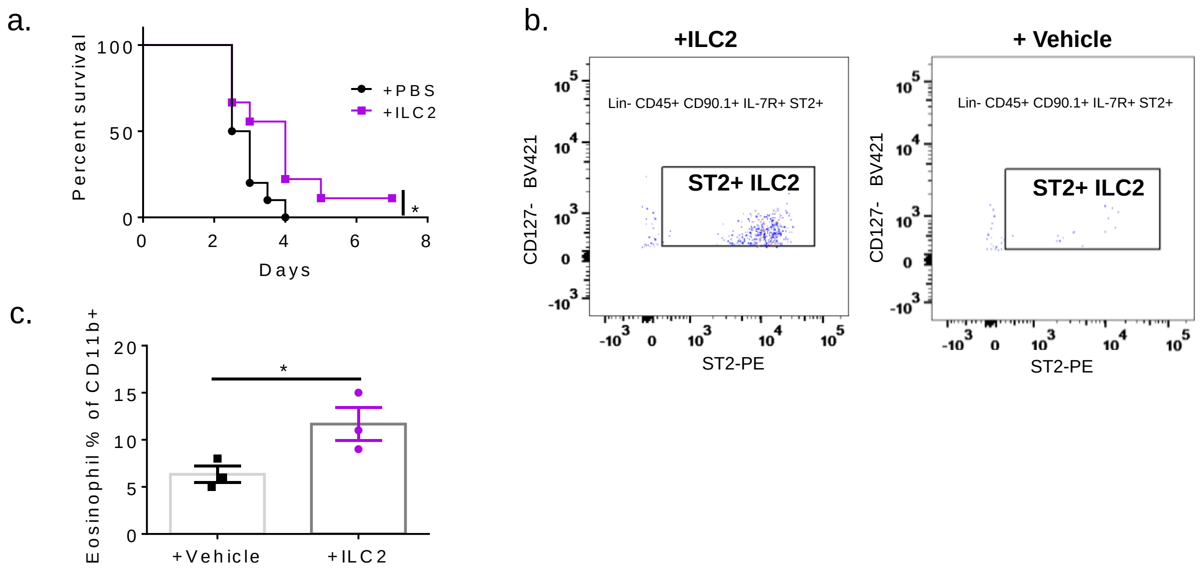

Supplementary Figure 9. Direct ex-vivo transfer of ILC2s protects from CDI-associated mortality and increases colon eosinophilia during CDI.

(***a***) Survival of direct *ex vivo* adoptive transfer study where sort purified ST2+ ILC2s were isolated from the MLN, colon, and spleen of IL-33 treated mice (CD90.1) and, without expansion, were directly transferred into Rag2$^{-/-}$γc$^{-/-}$ mice followed by R20291 infection. (***b***) Representative flow plot of donor ILC2s within the colon of Rag2$^{-/-}$γc$^{-/-}$ ILC2 recipients vs. control recipients and (***c***) frequency of colonic eosinophils within ILC2 vs. control recipients during infection (Day 4). (***a***) Comparison made by log rank test (*n*=10 mice per group) ***(c)*** Two groups compared by student T test (*n*=3 mice per group). Statistical significance is demarked as *P < 0.05, **P < 0.01, and ***P < 0.001. Error bar indicates SEM.

Supplementary Figure 10

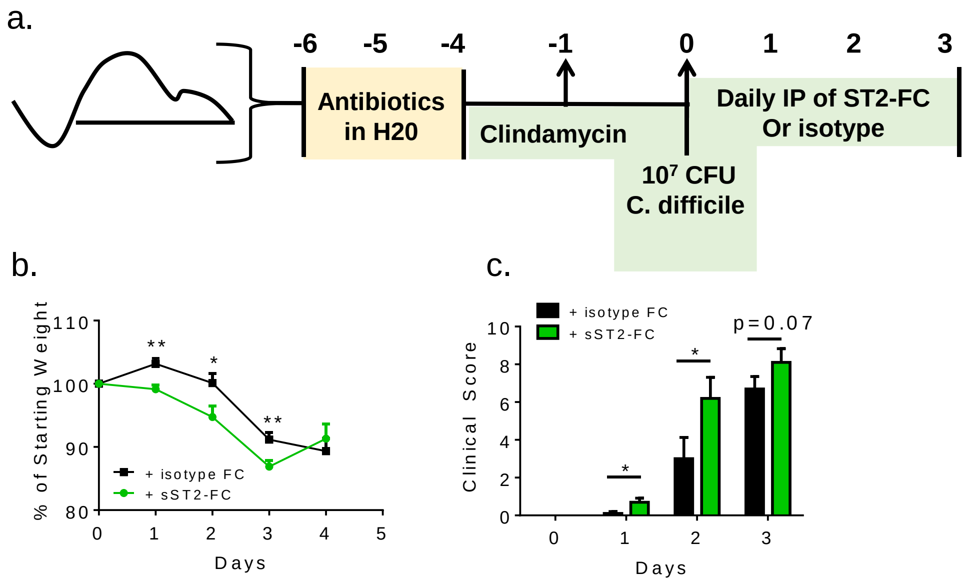

Supplementary Figure 10. Soluble ST2 increases *C. difficile* disease severity in mice.

(***a-c***) C57BL6 mice were infected with R20291 and subsequently given 4 injections of sST2-FC or isotype control by IP (Day 0-3). (***a***) Experimental design of sST2 treatment and *C. difficile* infection model. (***b***) Weight-loss and (***c***) clinical scores were assessed after treatment and infection. (***b,c***) n= 10 representative of 2 independent experiments and comparison made by student t test. Statistical significance is demarked as *P < 0.05, **P < 0.01, and ***P < 0.001. Error bar indicates SEM.

Supplementary Figure 11

a.

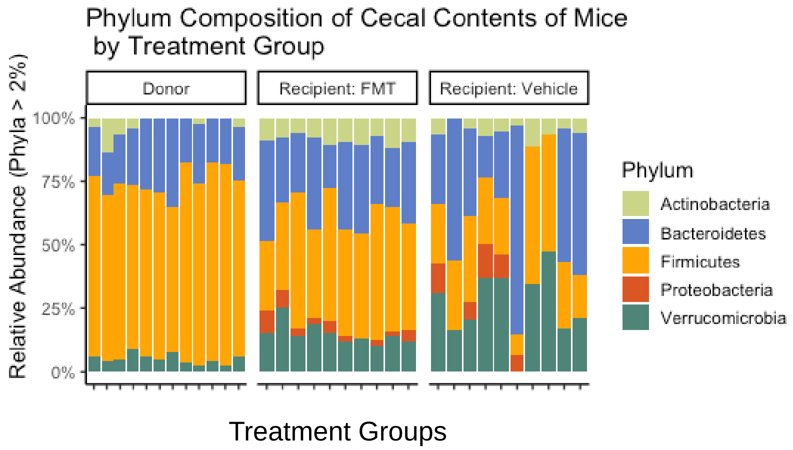

b.

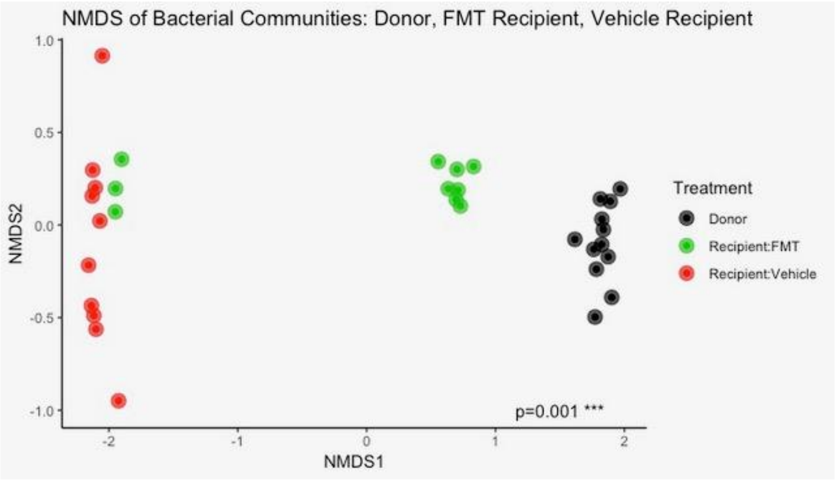

Supplementary Figure 11. FMT changes the gut microbiota after treatment.

Mice were treated with an FMT after broad-spectrum antibiotic cocktail used in infection model. 48 hours post- FMT, 16S rRNA sequencing was conducted. (***a***) Bar plot of relative phylum abundance of Donors vs. FMT recipients vs. vehicle controls. (***b***) NMDS plot of Bray-Curtis dissimilarity index from caecal samples of Donors vs. FMT and Vehicle recipient mice. (***b***) comparisons made using permutational multivariate analysis of variance (PERMANOVA) (n=10 mice per group). *P < 0.05, **P < 0.01, and ***P < 0.001.

**Supplementary Table 1.** Mortality risk of CDI + human patients based on sST2 levels.							
**Parameter**		Standard Error	Chi-Square	Pr > ChiSq	**Hazard Ratio (Mortality Risk)**	95% Hazard Ratio Confidence Limits	
**sST2**	>50%tile	0.44357	6.8323	**0.0090**	**3.188**	1.336	7.605
**Age**		0.01573	9.8886	**0.0017**	**1.051**	1.019	1.084
**Gender**		0.40698	0.7917	0.3736	1.436	0.647	3.189
**Race**		0.48369	0.0001	0.9921	1.005	0.389	2.593
**Charlson Index**		0.05366	0.8791	0.3484	1.052	0.947	1.168

[1] Correlation between ST2 levels in serum and survival assessed through Cox regression analysis.

[2] Mortality risk (hazard ratio) adjusted for age, sex, race, charlson comorbidity index.

Supplementary Table 2			
**Age Distribution**			
**Age**	**Rank for sST2**		**All**
	**≤ 50%tile (median)**	**> 50%tile (median)**	
**N**	**79**	**77**	156
**Mean**	60.56	62.01	61.28
**Std**	15.51	16.12	15.78
**Min**	22	17	17
**Q1**	50	54	52
**Median**	61	64	63
**Q3**	70	74	73
**Max**	95	88	95

[1] Age distribution of CDI + human patients analyzed for ST2 expression.

Supplementary Table 3						
**Gender and race demographic**						
	**Rank for sST2**				**All**	
	**≤ 50%tile (median)**		**> 50%tile (median)**			
	**N**	**%**	**N**	**%**	**N**	**%**
**Female**	81	46.9	79	51.9	160	49.4
**African American**	81	23.5	79	20.3	160	21.9

[1] Demographic distribution of CDI + human patients analyzed for ST2 expression.
