# Supplementary material for: IL-33 drives group 2 innate lymphoid cell-mediated protection during Clostridium difficile infection
Source: Nat Commun. 2019 Jun 20;10:2712. doi: 10.1038/s41467-019-10733-9 (PMC6586630; doi:10.1038/s41467-019-10733-9)
Supplement: Supplementary file 3 — Reporting Summary [file 41467_2019_10733_MOESM3_ESM.pdf]

nature research

Corresponding author(s): William A. Petri
Last updated by author(s): 2019/05/13

Reporting Summary

Nature Research wishes to improve the reproducibility of the work that we publish. This form provides structure for consistency and transparency in reporting. For further information on Nature Research policies, see Authors & Referees and the Editorial Policy Checklist .

Statistics

For all statistical analyses, confirm that the following items are present in the figure legend, table legend, main text, or Methods section.

n/a	Confirmed	
☐	☒	The exact sample size (*n*) for each experimental group/condition, given as a discrete number and unit of measurement
☐	☒	A statement on whether measurements were taken from distinct samples or whether the same sample was measured repeatedly
☐	☒	The statistical test(s) used AND whether they are one- or two-sided<br>*Only common tests should be described solely by name; describe more complex techniques in the Methods section.*
☐	☒	A description of all covariates tested
☐	☒	A description of any assumptions or corrections, such as tests of normality and adjustment for multiple comparisons
☐	☒	A full description of the statistical parameters including central tendency (e.g. means) or other basic estimates (e.g. regression coefficient) AND variation (e.g. standard deviation) or associated estimates of uncertainty (e.g. confidence intervals)
☐	☒	For null hypothesis testing, the test statistic (e.g. *F*, *t*, *r*) with confidence intervals, effect sizes, degrees of freedom and *P* value noted<br>*Give P values as exact values whenever suitable.*
☒	☐	For Bayesian analysis, information on the choice of priors and Markov chain Monte Carlo settings
☒	☐	For hierarchical and complex designs, identification of the appropriate level for tests and full reporting of outcomes
☐	☒	Estimates of effect sizes (e.g. Cohen's *d*, Pearson's *r*), indicating how they were calculated

Our web collection on statistics for biologists contains articles on many of the points above.

Software and code

Policy information about availability of computer code

Data collection

no software was used for collection of data

Data analysis

Software used in the study for 16S and Microarray Analysis:
• R
https://www.r-project.org/

For Microarray analysis:
1) Expression intensities were summarized, normalized, and transformed using Robust Multiarray Average algorithm
• Citation: Bolstad, B.M., Irizarry, R.A., Åstrand, M. and Speed, T.P., 2003. A comparison of normalization methods for high density oligonucleotide array data based on variance and bias. Bioinformatics, 19(2), pp.185-193.

2) For examining differential gene expression, a linear model was fit with empirical-Bayes moderated standard errors using the limma package in R.
• http://bioconductor.org/packages/release/bioc/html/limma.html

For Microarray Pathway analysis:
1) Qiagen Ingenuity Pathway Analysis
•https://www.qiagenbioinformatics.com/products/ingenuity-pathway-analysis/
•Krämer, A., Green, J., Pollard Jr, J. and Tugendreich, S., 2013. Causal analysis approaches in ingenuity pathway analysis. Bioinformatics, 30(4), pp.523-530.

2) Consensus Path DB
•http://consensuspathdb.org/

•Kamburov, A. et al. (2013) The ConsensusPathDB interaction database: 2013 update. Nucleic Acids Res.

For 16S Analysis Software used in this study:
Packages within R used for analysis
• DADA2
https://benjjneb.github.io/dada2/dada-installation.html
• Phyloseq
https://joey711.github.io/phyloseq/
• Vegan
https://cran.r-project.org/web/packages/vegan/index.html

For manuscripts utilizing custom algorithms or software that are central to the research but not yet described in published literature, software must be made available to editors/reviewers. We strongly encourage code deposition in a community repository (e.g. GitHub). See the Nature Research guidelines for submitting code & software for further information.

Data

Policy information about availability of data

All manuscripts must include a data availability statement. This statement should provide the following information, where applicable:

- Accession codes, unique identifiers, or web links for publicly available datasets
- A list of figures that have associated raw data
- A description of any restrictions on data availability

The microarray data generated in this study is available at the Gene Expression Omnibus under accession number GSE122013. Genes that met statistical significance in this study are listed in Supplemental Table 1. Raw sequence files for 16S V4 sequencing have been deposited in the Sequence Read Archive database under the project PRJNA521980. Other data from the findings of this study are available from the corresponding author upon request

Field-specific reporting

Please select the one below that is the best fit for your research. If you are not sure, read the appropriate sections before making your selection.

[x] Life sciences [ ] Behavioural & social sciences [ ] Ecological, evolutionary & environmental sciences

For a reference copy of the document with all sections, see nature.com/documents/nr-reporting-summary-flat.pdf

Life sciences study design

All studies must disclose on these points even when the disclosure is negative.

Sample size	Sample size was determined by a power of 90% to detect differences between intervention and control group for a p value less than 0.05.
Data exclusions	In human sST2 data, 7 observations with invalid time, censoring, or strata values were excluded from survival and cox-regression.
Replication	Results presented are representative of experiments repeated 2-3 times.
Randomization	Mice received from Jackson were randomized into different cages and each treatment group had at least two cages per group. Transgenic mice were cohoused to control for cage variability.
Blinding	Histopathology scoring was blinded and scored by 3 independent blinded scorers. Clinical scoring of mice was unblinded.

Reporting for specific materials, systems and methods

We require information from authors about some types of materials, experimental systems and methods used in many studies. Here, indicate whether each material, system or method listed is relevant to your study. If you are not sure if a list item applies to your research, read the appropriate section before selecting a response.

Materials & experimental systems

n/a	Involved in the study	
[ ]	[x]	Antibodies
[x]	[ ]	Eukaryotic cell lines
[x]	[ ]	Palaeontology
[ ]	[x]	Animals and other organisms
[ ]	[x]	Human research participants
[x]	[ ]	Clinical data

Methods

n/a	Involved in the study	
[x]	[ ]	ChIP-seq
[ ]	[x]	Flow cytometry
[x]	[ ]	MRI-based neuroimaging

Antibodies

Antibodies used	CD19-PerCPCY5.5, clone 6D5, biolegend 115533<br>GATA3-BV711, BD biosciences #565449, Clone (L50-823)<br>Anti-IFN-g Brilliant Violet® 605, [clone: XMG1.2] (Biolegend #505839)<br>IL-13-PEeFluore610, Clone eBio13A, Thermo fisher # 61-7133-82<br>BV421 Rat Anti-Mouse CD127 Clone A7R34, BD 566377<br>PerCP/Cy5.5 anti-mouse CD5 Antibody, clone 53-7.3, biolegend (100623)<br>PerCP/Cy5.5 anti-mouse CD3ε Antibody, clone 145-2C11 , biolegend catalog (100327)<br>PE/Cy7 anti-mouse FcεRIα Antibody, clone Mar1, Catalog (134317)<br>PerCP/Cy5.5 anti-mouse CD11c Antibody, clone n418, biolegend catalog (117327)<br>FITC anti-mouse CD90.2 Antibody, clone 30-h12, biolegend catalog (105305)<br>ROR gamma (t) Monoclonal Antibody (clone B2D), APC, eBioscience™ #17-6981-82<br>IL-33R (ST2) Monoclonal Antibody (RMST2-33), PE, eBioscience™ 12-9333-82<br>PE/Dazzle™ 594 anti-T-bet Antibody, clone 4b10, biolegend #644827<br>PE/Cy7 anti-mouse/human CD11b Antibody, clone m170, biolegend #101215<br>PE Rat Anti-Mouse Siglec-F Clone E50-2440, BD 552126<br>Brilliant Violet 421™ anti-mouse CD11c Antibody, clone n418, biolegend #117329<br>FITC anti-mouse Ly-6C Antibody, clone hk14, biolegend # 128005<br>APC/Cyanine7 anti-mouse CD45 Antibody Biolegend# 103115<br>PE/Cy7 anti-mouse Ly-6G Antibody, clone 1A8, biolegend # 127617<br>APC anti-mouse/human CD11b Antibody, clone m1/70, biolegend # 101211<br>Mouse IL-33 Antibody R and D systems, #AF3626<br>R & D, Human IL-33 Antibody Antigen Affinity-purified Polyclonal Goat IgG; Catalog Number: AF3625
Validation	Primary antibody used in this study for IHC:<br>1) R & D systems: Mouse IL-33 Antibody Antigen Affinity-purified Polyclonal Goat IgG Catalog Number: AF3626<br>Measured by its ability to neutralize IL¬33 induced proliferation in the D10.G4.1 mouse helper T cell line Schmitz, J. et al. (2005) Immunity 23:479. The Neutralization Dose (ND50) is typically 10-50 ng/mL in the presence of 0.25 ng/mL Recombinant Mouse IL¬33.<br>Reference:<br>Onda, H. et al. (1999) J. Cereb. Blood Flow Metab. 19:1279. 2. Baekkevold, E.S. et al. (2003) Am. J. Pathol. 163:69. 3. Schmitz, J. et al. (2005) Immunity 23:479. 4. Black, R.A. et al. (1989) J. Biol. Chem. 264:5323. 5. Xu, D. et al. (1998) J. Exp. Med. 187:787. 6. Lohning, M. et al. (1998) Proc. Natl. Acad. Sci. USA 95:6930. 7. Dinarello, C.A. (2005) Immunity 23:461. 8. Chackerian, A.A. et al. (2007) J. Immunol. 179:2551.<br><br>2) Measured by its ability to neutralize IL-33-induced proliferation in the D10.G4.1 mouse helper T cell line. The Neutralization Dose (ND50) is typically 0.75-3.0 µg/mL in the presence of 1 ng/mL Recombinant Human IL-33 and sub-optimal amounts of Mouse CD3ε Monoclonal Antibody.<br>References: 1. Onda, H. et al. (1999) J. Cereb. Blood Flow Metab. 19:1279. 2. Baekkevold, E.S. et al. (2003) Am. J. Pathol. 163:69. 3. Schmitz, J. et al. (2005) Immunity 23:479. 4. Black, R.A. et al. (1989) J. Biol. Chem. 264:5323. 5. Xu, D. et al. (1998) J. Exp. Med. 187:787. 6. Lohning, M. et al. (1998) Proc. Natl. Acad. Sci. USA 95:6930. 7. Dinarello, C.A. (2005) Immunity 23:461. 8. Chackerian, A.A. et al. (2007) J. Immunol. 179:2551.

Animals and other organisms

Policy information about studies involving animals; ARRIVE guidelines recommended for reporting animal research

Laboratory animals	Experiments were carried out using sex matched 8–12 week old C57BL6, ST2-/-, Rag2-/-, and Rag2-/-γc-/- mice. C57BL6 were purchased from Jackson Laboratory (#000664) and ST2-/- mice were obtained from Dr. Andrew McKenzie (Laboratory of Molecular Biology, Cambridge University, Cambridge, United Kingdom). Rag2-/- (RAGN12 EF) and Rag2-/-γc-/- (4111 EF) mice were purchased from Taconic Biosciences with an excluded flora.
Wild animals	Study did not involve wild animals
Field-collected samples	study did not involve field collected samples
Ethics oversight	All procedures were approved by the IACUC at the University of Virginia (protocol # 3769).

Note that full information on the approval of the study protocol must also be provided in the manuscript.

Human research participants

Policy information about studies involving human research participants

Population characteristics	Human Patients:<br>N=167<br>Mean age: 61.27<br>Std Age: 15.78<br>Gender: 49.4 % female<br>African American: 21.9%
Recruitment	Human samples were obtained from clinical samples otherwise been discarded. Samples not linked to protected health information.
Ethics oversight	Approved by UVA IRB.

Note that full information on the approval of the study protocol must also be provided in the manuscript.

Flow Cytometry

Plots

Confirm that:

- [x] The axis labels state the marker and fluorochrome used (e.g. CD4-FITC).
- [x] The axis scales are clearly visible. Include numbers along axes only for bottom left plot of group (a 'group' is an analysis of identical markers).
- [x] All plots are contour plots with outliers or pseudocolor plots.
- [x] A numerical value for number of cells or percentage (with statistics) is provided.

Methodology

Sample preparation	Colons were dissected longitudinally and rinsed in HBSS supplemented with 25mM HEPES and 5% FBS. Epithelial cells were separated from the lamina propria via a 40 min incubation with gentle agitation in dissociation buffer (HBSS with 15 mM HEPES, 5 mM EDTA, 10% FBS and 1 mM DTT) at 37°C. Next, the lamina propria tissue was manually diced using scissors and further digested in RPMI 1640 containing 0.17 mg/mL Liberase TL (Roche) and 30 µg/mL DNase (Sigma). Samples were digested for 40 minutes at 37°C with gentle shaking. Single cell suspensions were generated by passaging samples through a 100 µM cell strainer followed by a 40 uM cell strainer (both Fisher Scientific).
Instrument	Collection conducted on BD LSRFORTESSA
Software	All compensation and gating of flow cytometry data was conducted using FlowJo Software (https://www.flowjo.com/solutions/flowjo/)
Cell population abundance	Cell population abundance was calculated based on cell frequency and total cell numbers counted using count-bright beads (Thermo C36950).
Gating strategy	Cells were gated on fsc height vs. area to gate on singlets, Live dead Aqua to gate on live cells. For myeloid populations cells were gated on CD45+ cells followed by CD11B+ cells, and then further subsetted into eosinophils (SiglecF+ Ly6G-), Monocytes (Ly6G-Ly6C+), and neutrophils (Ly6G+ Ly6C+). Positive gates drawn based on FMO controls.<br><br>For ILC populations, lineage negative cells were gated out (Lineage cells (CD19+, CD3+, CD5+, CD11c+, CD11b+, FcεR+) then ILCs further identified by Ssclow, CD45+ CD90+ CD127+ ILCs following by transcription factor staining for T-bet (ILC1+), GATA3+ST2+ (ILC2), RorγT (ILC3). Positive and negative gates defined by FMO control for each stain.

- [x] Tick this box to confirm that a figure exemplifying the gating strategy is provided in the Supplementary Information.
