# Supplementary material for: IL-33 drives group 2 innate lymphoid cell-mediated protection during Clostridium difficile infection
Source: Nat Commun. 2019 Jun 20;10:2712. doi: 10.1038/s41467-019-10733-9 (PMC6586630; doi:10.1038/s41467-019-10733-9)
Supplement: Supplementary file 4 — Description of Additional Supplementary Files [file 41467_2019_10733_MOESM4_ESM.pdf]

Description of Additional Supplementary Files

File Name: Supplementary Data 1
Description: Genes dysregulated in response to CDT toxin expressing hypervirulent C. difficile.
